# The Interplay between Defensins and Microbiota in Crohn's Disease

**DOI:** 10.1155/2017/8392523

**Published:** 2017-01-26

**Authors:** Lorena Coretti, Alessandro Natale, Mariella Cuomo, Ermanno Florio, Simona Keller, Francesca Lembo, Lorenzo Chiariotti, Raffaela Pero

**Affiliations:** ^1^Institute of Endocrinology and Experimental Oncology (IEOS), National Research Council (CNR), 80131 Naples, Italy; ^2^Department of Molecular Medicine and Medical Biotechnology, University of Naples “Federico II”, 80131 Naples, Italy; ^3^Department of Pharmacy, University of Naples “Federico II”, 80131 Naples, Italy

## Abstract

Crohn's disease (CD) is a chronic inflammation of the intestinal mucosa, characterized by periods of acute recurrence and remission. Depending on the specific region affected, CD is classified as ileal CD or colonic CD. It is largely accepted that the intestinal microbiota is involved in the onset of the pathology. Indeed, a reduced immune tolerance to components of the intestinal commensal microbiota and inflammation of the intestinal barrier typifies patients with CD. Several studies have shown defective expression of intestinal antimicrobial peptides (AMPs) in patients with CD compared to controls, particularly defensins. A reduction in *α*-defensins is observed in ileal CD, while *β*-defensins are increased in colonic CD. In addition to an immunological basis, the disease is frequently associated with genetic alterations including mutations of NOD2 gene. Several therapeutic strategies to circumvent the dysfunction observed in CD are currently under investigation. These include the use of delivery systems to administer endogenous AMPs and the engineering of peptidomimetics that could ameliorate the severity of CD. In this review, the role defensins play in CD and the strategies aimed at overcoming bacterial resistance will be discussed.

## 1. Introduction

Crohn's disease (CD) is one of the principal types of inflammatory bowel disease (IBD) that can affect the intestinal mucosa. Although the etiology of the disease is still unclear, contributing factors include environmental elements, genetic mutations, and pathogenic infections. In genetically predisposed individuals, these factors are responsible for an aggressive and damaging immune response to microorganisms of the intestinal microbiota. CD can attack any part of the digestive tract, but in 50% of cases, it typically manifests in the gastrointestinal tract and can be classified, based on the specific region affected, as either ileal or colonic CD. Ileal CD is restricted to the ileum and accounts for 30% of cases, while colonic CD accounts for the remaining 20% of cases and may be particularly difficult to distinguish from ulcerative colitis (UC) [1].

The lowest incidence rates for CD are found in Asia and developing countries, with higher rates in the Western World. For example, the incidence of CD in Europe and North America is approximately 3 per 100,000 people. In recent decades, CD incidence has been relatively stable in the West, whereas it is becoming more prevalent in Asia [2].

The role of innate immunity in CD became clear after it was determined that* NOD2/CARD15* (Nucleotide-Binding Oligomerization Domain 2) was a susceptibility-associated gene.* NOD2/CARD15* acts as an intracellular sensor of pathogen/microbe-associated molecular patterns (PAMPs or MAMPs) [3, 4]. Recent studies have also identified a genetic association between CD susceptibility and autophagy genes, including* ATG16L1* (Autophagy-Related 16-Like 1) and* IRGM* (Immune-Related GTPase M) [5–7]. Genetic, immunological, and microbial studies have shown that the etiology of IBD involves a reduced tolerance to components of the intestinal commensal microbiota [8]. Because of the large number of enteric bacteria in contact with the intestinal mucosal surface and the potential threat of opportunistic invasions, a perfect homeostasis is central for the maintenance of the intestinal-microbial interface. This homeostasis is mediated by the integrity of the intestinal barrier and a functional immunotolerance to intestinal microbiota and luminal antigens.* NOD2*, the first gene identified for IBD susceptibility, represents an example of a mechanism by which inflammatory disease is propagated by a dysregulated response to the symbiotic microbiota [3, 4, 9]. The aim of this review is to discuss recent developments on the general hypothesis that Crohn's disease is caused by a defect in the defensin-mediated antibacterial mucosal barrier.

## 2. Microbiota in the Intestinal Mucosa and Dysregulation in CD

The human intestinal microbiota is dominated by four bacterial populations; about 90% belong to the phyla of Firmicutes and Bacteroidetes, while the remainder is comprised of the phyla of Proteobacteria (such as* Escherichia *and* Helicobacter*), Actinobacteria, and numerous viruses, protists, and fungi [10, 11]. Balance in the composition of gut microbiota is critical for the health of the host. Indeed, the microbiota contributes to a wide range of metabolic functions, including development, immune response, and nutrition [12].

Some microbial hydrolytic enzymes produced by members of the intestinal microbiota are important for the fermentation of nondigestible dietary fiber (polysaccharides, resistant starch, fibers of plant origin, and nondigestible oligosaccharides) [13]. The principal products of this fermentation are hydrogen, carbon dioxide, and short-chain fatty acids (SCFAs) such as butyrate and propionate [14]. SCFAs are an important energy source for the host, with a central role in lipogenesis and gluconeogenesis [15]. Moreover, they also have an important anti-inflammatory function due to the suppression of inflammatory cytokines [16].

The intestinal microbiota is therefore a key modulator of the immune system. For example, gut microorganisms play a role in the differentiation of different effector and memory T cell populations and are necessary to produce both proinflammatory (such as IL-17) and anti-inflammatory molecules (such as IL-10) [17].

Dysregulation of the intestinal microbiota (“dysbiosis”) has been reported in many patients with CD. For example, decreased complexity in commensal bacterial profiles and higher numbers of mucosa-associated bacteria have been shown to be associated with CD development [18]. Metagenomic studies have also shown some differences in the microbiomes of patients with CD and controls. The most interesting conclusions from these studies are as follows: (i) there is a quantitative decrease of several species of the Firmicutes and Bacteroidetes phyla in patients with CD. Among the Bacteroidetes,* Bacteroides fragilis (B. fragilis) *has shown important protective effects in mouse models of induced colitis [19]. Moreover, among the Firmicutes, a decrease in the number of* Faecalibacterium prausnitzii (F. prausnitzii)* was observed [20]. In mouse models of intestinal inflammation, oral administration of* F. prausnitzii *reduced inflammatory effects and helped to restore the normal microbiota composition [20]. Therefore, the decreased abundance of* B. fragilis* and* F. prausnitzii* could contribute to the intestinal inflammatory status in patients with CD; (ii) there is an increased level of the Proteobacteria and Actinobacteria phyla, including the family of Enterobacteriaceae, in patients with CD. In particular, the amounts of adherent-invasive* E. coli* (AIEC) were found to be higher in patients with lesions of the ileal mucosa. Studies have demonstrated the ability of AIEC to invade cells such as macrophages, replicate intracellularly, and stimulate the release of large quantities of TNF (Tumor Necrosis Factor), a proinflammatory factor [21, 22]; (iii) there is an increase in the abundance of bacteria penetrating the mucus layer in patients with CD. Some mucolytic bacteria such as* Ruminococcus gnavus* and* Ruminococcus torques* were observed in the intestinal epithelium of patients with CD. Therefore, the microbiota may have closer contact with the mucosa during CD [23].

## 3. Role of the Intestinal Membrane and Innate Immune Response

The intestinal barrier plays an important role in the functions of the immune system and although it allows the absorption of ions, water, and other nutrients, it behaves as a barrier to prevent the infiltration of bacteria through the mucosal surface. A layer of columnar epithelial cells provides the first line of defense against microorganisms entering the intestinal lumen. More than 80% of these cells are enterocytes, while the remaining are enteroendocrine, goblet, or Paneth cells [24]. In many studies, it has been shown that there is a relationship between variation in intestinal permeability and the status of CD disease. Permeability was increased during acute CD phases while it was decreased during remission phases [25, 26]. The increased intestinal permeability could also favour bacterial translocation through the mucosa [27]. Moreover, the intestinal epithelial surface is covered by a mucus layer composed primarily of mucins, heavily glycosylated proteins secreted by goblet cells. A thin and discontinuous mucus layer in small intestine prevents direct contact of bacteria with the epithelium and Peyer's patches [28]. In the large intestine, the mucus layer is dense and composed by an inner and outer mucus layer [29]. The outer mucus layer contains a large number of microbes and appears to be an amenable habitat for commensal bacteria. However, the inner adherent mucus layer contains a very low number of microbes and forms a protective zone adjacent to the epithelial surface [30]. The low bacterial abundance in the inner layer is likely due to the presence of antimicrobial proteins (AMPs). These AMPs, which are secreted by epithelial cells and retained in the mucus layer, promote bacterial killing by targeting the integrity of bacterial cell walls [31]. Therefore, the action of some antibacterial proteins (such as RegIII*γ*, defensins, and IgA) is important in preventing direct contact between the epithelial layer and microorganisms. The diffusion of undesired substances and dysregulated synthesis of AMPs could result in decreased protection and an increased permeability to bacteria in the intestinal barrier. This would have consequent effects on local inflammation [32, 33].

Changes in this process, which could contribute to abnormal immune responses, have been observed in CD. For example, dysregulated IgA production has been demonstrated in patients with CD. Normally, IgA immunoglobulins are secreted by B cells in the intestinal lamina propria and maintained in the mucus layer. The production of IgA is regulated by dendritic cells that recognize bacteria present in the intestinal epithelium. This allows B cells in Peyer's patches of the ileum to produce IgA specific for intestinal bacteria [34]. Moreover, IgA regulates the composition and density of intestinal commensal bacteria [35]. Conversely, in patients with CD, IgA-mediated immune response is replaced by an IgG-mediated response that has numerous effects on the local microbiota [36].

Another immune defense mechanism against intracellular pathogens is autophagy. Autophagy is the biological process of cellular components degradation by lysosomal vesicles derived from the endoplasmic reticulum. A recent study found several polymorphisms in autophagy genes implicated in CD disease:* ATG16L1* (Autophagy-Related 16-Like 1),* IRGM* (Immune-Related GTPase M),* ULK1* (Unc-51 like autophagy activating kinase 1),* PTPN2 *(protein tyrosine phosphatase nonreceptor type 2), and* LRRK2 *(leucine-rich repeat kinase 2) [37]. Because* NOD2* recruits and interacts with* ATG16L1*, the presence of the* ATG16L1* or* NOD2 *risk variants could result in a defective response to pathogenic species [38–40]. Patients with CD who are homozygous for the* ATG16L1 *allele exhibit several structural abnormalities in Paneth cells such as disorganized granule compartments, fewer granules, and a lack of lysosomes in the ileal mucus layer [39]. Paneth cells are specialized epithelial cells that normally regulate the secretion of AMPs. A defect in the autophagy process in patients with CD could lead to uncontrolled bacterial proliferation and may consequently cause chronic inflammation.

## 4. Defensins: The Main Antimicrobial Proteins

Among the AMPs, there are peptides termed defensins that are an important component of innate immunity. Ten defensins have been identified in humans and classified through structural differences into a family of six *α*-defensins (HD) and a family of four *β*-defensins (HBD). The *α*-defensins are primarily secreted by Paneth cells, neutrophils, and certain macrophage populations of the small intestine. *β*-defensins are typically secreted by epithelial cells. Alpha-defensins HD1-4 are also called human neutrophil peptides (HNP) and participate in systemic innate immunity, while HD5 and HD6 contribute to innate defense of the GI mucosal surface [41].

In the gastrointestinal tract, the function of these peptides is to form micropores in bacterial membranes, causing an efflux of ions and nutrients, loss of the structure, and eventual cell collapse [41]. Human defensins represent the main family of bacterial-membrane disrupting proteins and act through several molecular mechanisms [42]. One of these mechanisms includes the processing of inactive pro-*α*-defensins by trypsin in humans (matrix metalloproteinase 7 (MMP-7) in mice). This enzyme induces a conformational change that forms three sets of disulfide bonds between cysteine pairs in the protein. Newly formed *α*-defensins form a dimeric structure that produces a positively charged surface spatially separated from hydrophobic regions, facilitating insertion into negatively charged microbial membranes [43]. Other mechanisms employed do not directly target and kill bacteria. For example, HD6 oligomerizes forming long fibrils that “capture” bacteria and prevent their passage through the intestinal epithelium [44].

Defensins can also protect against pathogen colonization. *MMP*7^−/−^ mice have strong susceptibility to oral administration of* Salmonella *Typhimurium, while mice that overexpress* HD5* show greater resistance than wild-type mice. This suggests that *α*-defensins are fundamental for mucosal protection against intestinal bacterial pathogens [45, 46]. Moreover, mice that that do not express *α*-defensins (*MMP*7^−/−^) show distinct differences in their intestinal microbiota compared to mice that express *α*-defensins normally (wt). It is therefore likely that defensins may regulate the composition and density of bacterial communities in the digestive lumen [47].

## 5. Defensins: Regulation of Expression, Secretion, and Activity in the Small Intestine

The secretion and expression of epithelial defensins are controlled by several transcriptional and posttranslational mechanisms. The expression of some *α*-defensins and *β*-defensins is regulated in a microbial-independent manner, through the expression of proinflammatory cytokines including IL-1*β* (HD5, HD6, or HBD1). They can also be regulated in a bacterial-dependent way (HBD2) due to pattern recognition receptors (PRRs) of the immune system [41, 48, 49].

Biosynthesis of defensins is activated by receptors that recognize extracellular and intracellular bacterial components. These include toll-like receptors (TLRs) and NOD2 receptors [50, 51]. After binding specific conserved microbial molecules, TLRs activate pathway cascades that in turn activate nuclear factor kB (NFkB). This promotes the transcription of proinflammatory cytokines and AMPs [52]. Although the activation mechanism of* NOD2 *has not yet been elucidated, some studies have shown that activation is dependent on muramyl-dipeptide (MDP), a peptidoglycan component that is present in both gram-positive and gram-negative bacteria [53]. NOD2 receptors have important roles in increasing the bactericidal activity of Paneth cells and modulate the composition of the microbiota in the small intestine [54]. Alpha-defensins are stored as inactive propeptides within epithelial cell secretory granules and are proteolytically processed by trypsin to produce mature active proteins [55, 56]. The process of secretion is controlled by bacterial signals. In the small intestine, these signals likely originate from live bacteria or bacterial products such as LPS and act directly on Paneth cells to induce degranulation. Recent studies have suggested that Paneth cells also degranulate in response to cytokines secreted by immune cells [57]. Finally, proteins of the autophagy pathway also regulate granule exocytosis in Paneth cells, as previously described.

The mechanisms regulating the synthesis of *β*-defensins are still unclear. As to their activity, it has been demonstrated that HBD1 has minimal antimicrobial activity in the presence of oxidizing conditions. However, in the presence of a reducing environment that mimics the conditions of the intestinal lumen, there are conformational changes in HBD1 that allow it to elicit a potent antimicrobial activity [58].

## 6. Defensins and Crohn's Disease

CD is characterized by a chronic inflammation that can affect any part of the gastrointestinal tract, although the ileum and colon are the regions most commonly affected. Research into the mechanisms underlying this disease has found that CD likely develops due to impaired antimicrobial activity of the intestinal mucosa against components of the microbiota. Commensal and potentially pathogenic bacteria can infiltrate the intestinal epithelium, causing the chronic inflammation that is typical of the disease. Reduced expression of defensins was also found in patients with CD; ileal CD is characterized by a decrease in the abundance of certain *α*-defensins, while certain *β*-defensins are implicated in colonic CD. Among the factors that contribute to impaired production of defensins are mutations in the* NOD2* gene, polymorphisms in the promoters of defensin genes, and defects in the Wnt pathway. Because of the differences between ileal and colonic CD, it is necessary to discuss them separately.

### 6.1. Defensins in Ileal Crohn's Disease

Wehkamp et al. have demonstrated that ileal CD is associated with a marked decrease in HD5 and HD6 expression in Paneth cells, compared to controls [59]. This relative decrease in AMPs reduces the antibacterial activity of the gut mucosa and consequently allows the penetration of pathogenic bacteria [60].

The same researchers have also proposed an association between the NOD2 receptor expressed in intestinal epithelial cells and the regulation of *α*-defensin expression. Patients with ileal CD and NOD2 mutations show decreased amounts of HD5 and HD6 mRNA in affected tissue, compared to patients without similar mutations [61]. Depending on ethnicity, a variable percentage of patients affected by CD have a mutation in the* NOD2 *gene [62]. Studies have shown that three* NOD2* alleles are associated with ileal CD susceptibility, the missense mutations Arg702Trp and Gly908Arg, and the frameshift mutation Leu1007fsincC. Another outcome was that the degree of inflammation was not associated with reduced HD5 [63]. These results suggest that a loss-of-function mutation in the* CARD15 *gene encoding NOD2 was responsible for the reduced antimicrobial activity observed at the mucosa [64]. However, some studies showed that the NOD2 mutation alone may be not sufficient to explain the HD5 decrease in IBD [65]. It has been proposed that reduction of alpha-defensin expression is mainly due to loss of surface epithelium as a consequence of inflammatory changes. [65].

Another mechanism that may be involved in the reduced levels of *α*-defensins in ileal CD is epithelial stem cell differentiation meditated by Wnt signaling. There are two distinct Wnt signaling mechanisms: the canonical and noncanonical pathways. The canonical pathway does not require the presence of *β*-catenin in order to be activated. The noncanonical pathway is activated by *β*-catenin which, when translocated into the nucleus of Paneth cells, interacts with T cell Factor 4 (TCF4). This resulting *β*-catenin-TCF4 complex binds to DNA in the nucleus, activating a suite of genes that include the defensins [66]. Therefore, deficiency in TCF4 is involved in the Paneth cell *α*-defensins reduction [67]. Furthermore, polymorphisms in the gene encoding the low-density lipoprotein receptor-related protein 6 (LRP6), another component of the Wnt signaling pathway, has been associated with ileal CD [68]. Therefore, mutations in NOD2, TCF4, and LRP6 could lead to decreased alpha-defensin production and consequent inflammation. Interestingly, inadequate Wnt ligand stimulation by monocytes induces defective Paneth cell-mediated innate immunity providing an additional mechanism in CD [69]. Because defects of Paneth cell function may be reversed by Wnt ligands, this mechanism was suggested as a potential therapeutic target for this disease [69].

Finally, hypomorphic alleles of the gene encoding the X-box-binding protein 1 (XBP1) are also associated with intestinal inflammation. XBP1 is important for the development of secretory cells and mice lacking XBP1 exhibit Paneth cell dysfunction and associated intestinal inflammation [70].

### 6.2. Defensins in Colonic Crohn's Disease

Unlike ileal CD, the levels of *α*-defensins remain unchanged in colonic CD, although significant differences in the expression of *β*-defensins are observed. While HBD1 is constitutively expressed, HBD2 can be induced by both inflammatory stimuli and signals derived from pathogenic or probiotic bacteria, including* Escherichia coli* strain Nissle and certain lactobacilli [71, 72].

Colonic CD is associated with increased secretion of HBD2 but also low HBD1 levels [73]. In a noninflammatory state, the expression of inducible *β*-defensins is low, but expression is significantly increased during inflammation generated in response to proinflammatory molecules, such as IL-1 [74]. Wehkamp et al. have confirmed this by showing that HBD2 is more abundant in patients with CD and UC [75]. Moreover, there was increased expression of HBD2 in inflamed tissues compared to noninflamed sites from the same patient [75, 76]. Two other inducible *β*-defensins, HBD3 and HBD4, were found to be only minimally expressed in control patients and no significant changes in patients with CD were observed [77]. To explain the impaired expression of some *β*-defensins that occurs in colonic CD, a decrease in *β*-defensins copy number on chromosome 8 was hypothesized. Fellermann et al. found significant differences between control groups and patients with CD; controls had a median of four copies while colonic patients with CD had a median of three [78].

In the intestine, HBD1 is partially under the control of the nuclear receptor peroxisome proliferator activated receptor *γ* (PPAR-*γ*), an essential mediator of intestinal homeostasis [79]. Abnormal PPAR-*γ* production results in reduced antimicrobial activity of the mucosa. Moreover, the single nucleotide polymorphism (SNP) rs1800972 in the HBD1 promoter was also shown to have a protective role in colonic CD development and in other clinical contexts, including oral infections and HIV infection. SNP rs1800972 is also involved in the transcriptional expression of HBD3 [80–82].

## 7. Future Perspectives

Understanding the mechanisms underlying CD is necessary for the future development of new therapies. There are now new strategies that aim to reinforce intestinal barrier function and restore the microbiota balance.* E. coli *strain Nissle 1917,* Lactobacillus, *and other known probiotics may have a common feature of activating AMP induction. This may play an important role in helping the mucosa prevent bacterial invasion [71, 72]. The administration of endogenous AMPs as novel therapeutic antibiotics also has potential but it is necessary to first overcome some obstacles. These include the fact that although AMPs do not readily induce bacterial resistance, they have several specific properties (proteolytic degradation by microbial and host enzymes, toxicity to eukaryotic cell membranes) that make developing them into therapeutics difficult [83, 84].

Several of these issues may be resolved through protein engineering. For example, the creation of AMP variants that are resistant to enzymatic digestion will allow the killing of bacteria directly through interactions with bacterial membranes [85]. Another strategy to improve the therapeutic potential of AMPs may be the engineering of peptidomimetics. These synthetic molecules mimic AMP structure but are constructed with an altered number of charged amino acids, reducing hydrophobicity and thus decreasing toxicity to host cells [86]. Many of these peptidomimetics have potent bactericidal activity against drug-resistant bacteria [87]. To improve the delivery efficiency and reduce nonspecific cytotoxicity, it may also be necessary to package natural AMPs or peptidomimetics in carrier nanoparticles [88]. With these and other approaches, researchers are now working on developing several new therapeutic molecules targeting CD.

## 8. Conclusions

AMPs, in particular defensins, are important for stabilizing the composition of the intestinal microbiota. They perform this action by preventing bacterial overgrowth and the invasion of the epithelium by potentially pathogenic bacteria, therefore facilitating normal function of the intestinal tract.

As we have outlined, alterations in expression of defensins have negative effects on the microbiota and can cause an inflammatory state. Defensin expression is intrinsically altered in IBD and it may play a significant role in its pathogenesis. In particular, ileal CD is associated with a lack of Paneth cells, with a consequent reduction in HD5 and HD6. Colonic CD is associated with a reduction in constitutive HBD1 and an increase in inducible HBD2. Additional genetic factors have also been identified, demonstrating that most patients with CD have mutations in the* NOD2* gene. Although these* NOD2* mutations play a role in the etiology of CD, several studies have demonstrated that they alone are not sufficient for the development of the disease. Restoring normal expression of AMPs may rebalance the intestinal microbiota and ameliorate intestinal inflammation. Therefore, future studies are needed to develop new strategies to identify and administer potentially therapeutic molecules to patients with CD.
